# Seborrheic Keratosis Caused by Human Papillomavirus Type 20 Ameliorated by Zinc Oxide Ointment

**DOI:** 10.3390/clinpract13020033

**Published:** 2023-02-24

**Authors:** Makoto Kondo, Yoshiaki Matsushima, Takehisa Nakanishi, Shohei Iida, Koji Habe, Keiichi Yamanaka

**Affiliations:** Department of Dermatology, Mie University Graduate School of Medicine, 2-174, Edobashi, Tsu 514-8507, Japan

**Keywords:** HPV20, zinc transport protein-1, zinc, seborrheic keratosis, gene mutation

## Abstract

A 91-year-old woman visited our department with scattered small nodule lesions and multiple pules or plaques with a stuck-on appearance. The lesions were intractable and resistant to several treatments. Immunodeficiency was excluded by examinations including a CT scan, white blood cell (WBC) counts, natural killer and neutrophil function assays, and IgG titers against human papillomavirus (HPV) 20. HPV20 was identified using the PCR method. The finding of the skin biopsy showed an irritated type of feature of seborrheic keratosis. Additionally, immunohistochemical staining of the lesion revealed that both TNF-α and IFN-ɤ were produced at the skin lesions. The patient‘s serum zinc level was slightly low. We noticed that zinc deficiency has been reported to decrease the cytotoxic activity of natural killer cells, which play an important role in eliminating virus-infected cells and tumor cells. Finally, zinc oxide ointment was found to improve the lesions dramatically. HPV20 causes tumors only in immunodeficient patients or in patients with epidermodysplasia verruciformis (EV). In EV, EVER1- or EVER2-encoding membrane proteins, of which are related to zinc transport protein-1 expressed on the membrane of the endoplasmic reticulum, were mutated, leading to increased susceptibility to various viral and bacterial infections due to the decreased intracellular zinc concentration. We speculated that the reduction in local zinc concentration was ameliorated by using zinc oxide ointment, resulting in the recovery from HPV20 infection.

## 1. Introduction

Human papillomavirus (HPV) is divided into more than 100 known HPV genotype groups, and a long-lasting high-risk HPV infection leads to cancer, including the cervix, vulva, vagina, penis, anus, and mouth, unless adequate treatment for HPV is applied. HPV20 belongs to genus beta papillomavirus and is categorized into a low-risk HPV type that causes tumors only in immunodeficiency patients or epidermodysplasia verruciformis (EV) patients. EV patients have mutations in zinc transport protein (ZnT)-1-related genes. The immune system works with various immune cells to protect against bacterial and viral infections and to destroy cancerous cells. Zinc plays an important role in the immune system. Thus, zinc is an essential element for normal cell structure and physiology. Its deficiency causes growth retardation, neuronal degeneration, and immunodeficiency. EV patients develop seborrheic keratosis (SK), and HPV has been detected in 42 of 55 (76%) SK skin lesions [1]. We, here, reported a case where HPV20-related SK was ameliorated by the use of zinc oxide ointment. We will discuss the mechanism of onset and treatment options.

## 2. Case Report

**Figure 1:**
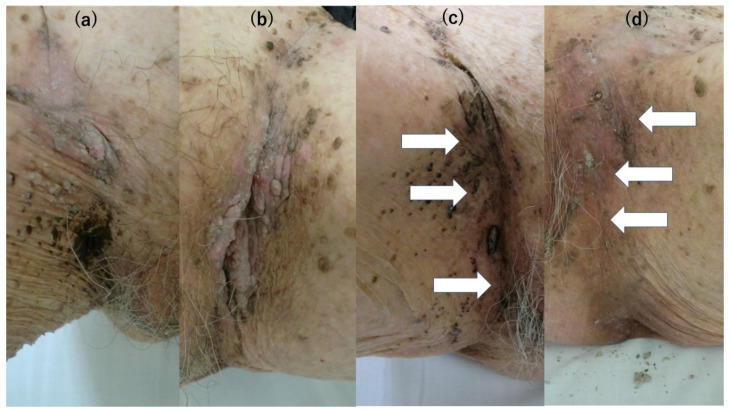
(**a**): SK developed at the left groin area along with the hip joint line. (**b**): SK in a part of the plaques developed at the right groin area. (**c**): SK lesion at the left groin area has recovered. The black nodules remain, but the white arrows indicate that the pinkish nodules have disappeared. A total of 90% of the areas of SK initially observed before and after the application of zinc oxide ointment disappeared. (**d**): SK lesion at the right groin area has recovered. White arrows indicate that the nodules and plaques have almost all disappeared.

A 91-year-old woman with a history of cerebral infarction and hypertension visited our department with small nodular lesions that developed gradually in the groin bilaterally over few months (Figure 1a,b). This patient was in a long-term nursing home, but there was no impairment in her ability to carry out her daily tasks. We treated the lesions with moisturizer by using Vaseline every day and with liquid nitrogen cryotherapy four times every two weeks; however, the number and sizes of grey and pinkish nodules and plaques remained unchanged. Therefore, we performed laboratory investigations and a whole-body CT scan because we suspected that acanthosis nigricans, internal organ malignancy, or immunodeficiency could be underlying causes. The CT scan showed no abnormal features, and the laboratory data on immunization were all within normal ranges: white blood cells 7200/μL (neutrophils 77.2%, lymphocytes 16.8%), natural killer cell activity: E/T ratio 10.8% (normal range: 8.9–29.5%) of effector cell: target cell = 10:1, neutrophils phagocytosis function of 52.2% (normal range: 40–80%), and sterilizing function of 99.9% (normal range: >70%). Laboratory data of liver function showed AST 16 IU/l (normal range: 10–35 IU/l) and ALT 11 IU/l (normal range: 10–35 IU/l), but that of renal function was slightly decreased with BUN 17.8 mg/dl (normal range: 9.0–22.0 mg/dl), Cre 0.83 mg/dl (normal range: 0.40–0.80 mg/dl), and eGFR 48.2 (normal range: 60.0–1000). Hemoglobin A 1c (HbA1c) was normal at 4.7% (normal range: 4.7–6.2%).

**Figure 2:**
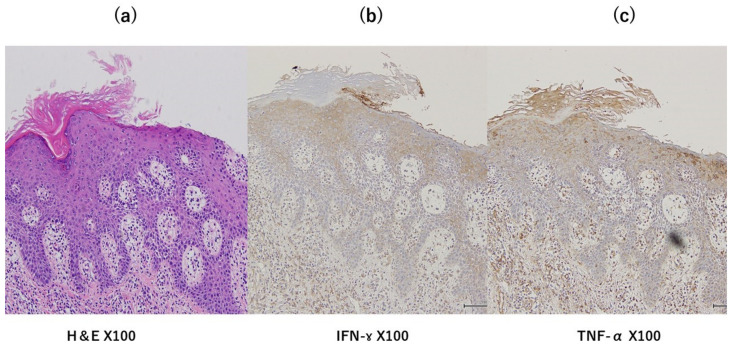
(**a**): The hematoxylin and eosin (HE) stain showed acanthotic proliferations as the irritated-type feature (scattered keratinocyte apoptosis and dyskeratosis, spongiosis, scale crust, and parakeratosis in the stratum corneum) of SK. (**b**): The INF-ɤ stained strongly in the epidermis and stained on mononuclear inflammatory cells for attacking SK. (**c**): TNF-α was stained weekly in epidermis and inflammatory cells compared with INF-ɤ.

Skin biopsy excluded malignancy tumor-like verrucous carcinoma and revealed seborrheic keratosis (SK) (Figure 2a). We tried to identify the type of human papillomavirus (HPV) because we suspected that the lesions were caused by a high-risk HPV type. The DNA from the SK was extracted using QIAamp DNA mini kit^®^ (Qiagen, German town, MD, USA). The HPV gene was evaluated using nested PCR, according to a previous report [2]. The reaction mixture was placed in a thermal cycler (ASTEL GeneAtlas 485, Fukuoka, Japan), where it underwent 5 cycles of denaturation (95 °C, 60 s), annealing (50 °C, 90 s), and extension (72 °C, 120 s); then, 35 cycles of denaturation (95 °C, 60 s), annealing (55 °C, 60 s), and extension (72 °C, 120 s). The sequences of the amplified PCR products were analyzed using Eurofins Genomics (Tokyo, Japan). Both the first and second PCRs were performed under the same conditions. The amplified fragment was identified as HPV20 by 100% hit to sequences deposited on GenBank (Accession number: KY9695393.1). However, HPV20 is a low-risk HPV type that causes tumors only in patients with immunodeficiency. Immune staining for IFN-ɤ and TNF-α on the skin biopsy specimen from the SK were positive for IFN-ɤ and TNF-α, especially in the epidermis (Figure 2b,c). An enzyme-linked immunosorbent assay (ELISA), using a recognized synthetic peptide that was specific for HPV20, showed normal ranges of IgG titers against HPV20. Therefore, we speculated that she had sufficient immunity against HPV20. We tried several treatment options such as Vaseline and four times of liquid nitrogen cryotherapy without improvement. Finally, a daily 20% concentration of zinc oxide ointment treatment drastically improved the SK lesions after 4 months (Figure 1c,d). Her serum zinc concentration was slightly decreased to 74 μg/dL (normal: 80–130 μg/dL). Later, the patient was found to have transverse colon cancer, but due to her advanced age, the patient was placed under observation and died one year later. After the diagnosis of the cancer, the application of zinc oxide ointment once a day to both inguinal areas was continued and no recurrence of SK was observed.

## 3. Discussion

HPV has been detected in SK skin lesions [1,3]; however, the detection ratio is as low as 1.9% and it is not a concern in healthy volunteers [4]. It had been reported that zinc treatment for 3 months reduced the risk of persistence of HPV infection and progression from baseline cytology [5]. In our case, the topical application of zinc for 4 months reduced the lesions of SK tumors by about 90% before and after zinc application. The results of them suggested that the zinc supplement increases the rates of HPV clearance and resolution of pre-existing SK lesions. Additionally, a low-risk type of HPV20 infection is clinically significant in epidermodysplasia verruciformis (EV) patients [6]. EV patients have mutations in either of two genes (EVER1 and EVER2) [7] that encode a membrane protein, of which forms a complex with a zinc transport protein (ZnT)-1 expressed on the membrane of the endoplasmic reticulum [8]. Mutations in these genes lead to infections with specific types of HPV of genus β (HPV5, 8, 9, 12, 14, 15, 17, 19–25, 36–38, 47, 49, etc.), bacterial infection, and cancer due to decreased intracellular zinc levels. Zinc deficiency is not only a hereditary deficiency such as EV, but it is also in various nutritional conditions (e.g., low nutrition, high-calorie infusion, hemodialysis, drug therapy, etc.) and physiological conditions. Zinc deficiency has also been reported to decrease the cytotoxic activity of natural killer cells, which play an important role in eliminating virus-infected cells and tumor cells [9]. Additionally, it has been reported that zinc supply suppresses the development of autoimmune diseases in mice [10,11]. Thus, zinc is essential for normal cell structure and physiology. Its deficiency causes growth retardation, neuronal degeneration, and immunodeficiency. It has been reported that the amount of zinc in the body decreases with aging. The patient in this case had slightly decreased serum zinc levels. Although the actual intracellular zinc was not measured, we suspect that it was quite low. In addition to the homeostasis of total zinc levels in serum, the importance of intracellular zinc ions in cellular responses has become clear. Zinc plays a role as a signaling molecule for cell-to-cell communication [12,13]. We excluded the possibility of an underlying gene mutation because of the late onset in the current case. The patient recovered from intractable SK after using zinc oxide ointment. We speculated that the patient recovered due to an improvement in local zinc level. It has been reported that the amount of zinc in the body decreases with aging; considering the super-aging society that the world is facing, it is important to continue pursuing studies regarding the relationship between zinc deficiency and the immune system.

## 4. Conclusions

In conclusion, we reported a rare case of HPV20-related SK of the groin that may have been associated with a reduced local zinc concentration. Zinc oxide ointment can be a treatment option for SK.

## Data Availability

All data that support the findings of this study are included in this article.
